# Vandetanib-Induced Hyponatremia and Torsades De Pointes: A Case Report

**DOI:** 10.7759/cureus.24556

**Published:** 2022-04-28

**Authors:** Shoaib Ashraf, Niel Shah, Muhammad Saad, Abhilasha Jyala, Timothy J Vittorio

**Affiliations:** 1 Internal Medicine, BronxCare Health System/Icahn School of Medicine at Mount Sinai, New York City, USA; 2 Cardiology, BronxCare Health System/Icahn School of Medicine at Mount Sinai, New York City, USA; 3 Cardiovascular Disease, BronxCare Health System/Icahn School of Medicine at Mount Sinai, New York City, USA

**Keywords:** vandetanib, long qt syndrome, ventricular tachycardia, torsades de pointes, hyponatremia

## Abstract

Medullary thyroid cancer (MTC) is a neuroendocrine tumor of the parafollicular cells of the thyroid gland. The prognosis is very poor in patients with advanced MTC. Vandetanib was approved for advanced MTC after randomized control trials showed that it had therapeutic efficacy and considerably prolonged progression-free survival. Vandetanib therapy is associated with serious cardiovascular side effects including hypertensive crisis and arrhythmias due to prolonged QTc. We present a case of an 83-year-old female with advanced metastatic MTC who is under treatment with vandetanib 300 mg/day and developed medication-related hyponatremia, QTc prolongation, ventricular fibrillation (VF), and torsades de pointes (TdP). Her vandetanib therapy was held. Subsequently, she did not show recurrences of TdP. This is the second such case report in the literature.

## Introduction

Tyrosine kinase inhibitors (TKIs) are used as chemotherapy because they are essential in modulating growth factor signaling [1]. Vandetanib is a TKI that targets the epidermal growth factor receptor (EGFR), vascular endothelial growth factor (VEGF) receptor 2, and the proto-oncogene protein RET. It is considered a promising treatment and an effective therapy for certain malignancies [2]. Several undesired side effects of TKIs are reported, making them less desirable in a particular population. TKIs commonly cause anemia, neutropenia, thrombocytopenia, skin toxicity, edema, hypothyroidism, hypertension, diarrhea, nausea, and vomiting [3]. Cardiac side effects are less common but are associated with many TKIs, including dasatinib, imatinib, sunitinib, nilotinib, sorafenib, and lapatinib. These cardiac undesired side effects can be subacute, acute, or chronic. Subacute and acute cardiac manifestations are commonly present as QT-interval changes and abnormalities in ventricular repolarization [4]. Medication-induced QT prolongation is associated with torsades de pointes (TdP), a life-threatening arrhythmia that can lead to sudden death [5]. We present an interesting case of TdP in a patient with medullary thyroid cancer (MTC) who was on treatment with vandetanib. The patient was also noticed to have severe hyponatremia that we attribute to vandetanib's use.

## Case presentation

An 83-year-old Caucasian female with metastatic MTC, diet-controlled diabetes mellitus, and hypertension was brought to the emergency department after a witnessed episode of sudden transient loss of consciousness. The patient also reported generalized weakness and dry mouth for the past few months. She had no prior history of seizure disorder, syncope, or familial history of syncope, cardiac arrest, or sudden cardiac death. The patient had an eight-year history of MTC and underwent a total thyroidectomy together with radical neck dissection and adjuvant radiation. Despite initial treatment, she experienced metastatic disease involving the lungs, liver, and bones. The biochemical analysis showed calcitonin levels of 7197 pg/mL (normal value < 5 pg/mL) and carcinoembryonic antigen (CEA) 195 ng/mL (normal value < 5 ng/mL). She was subsequently started on vandetanib 300 mg daily six months before the presentations.

**Figure 1:**
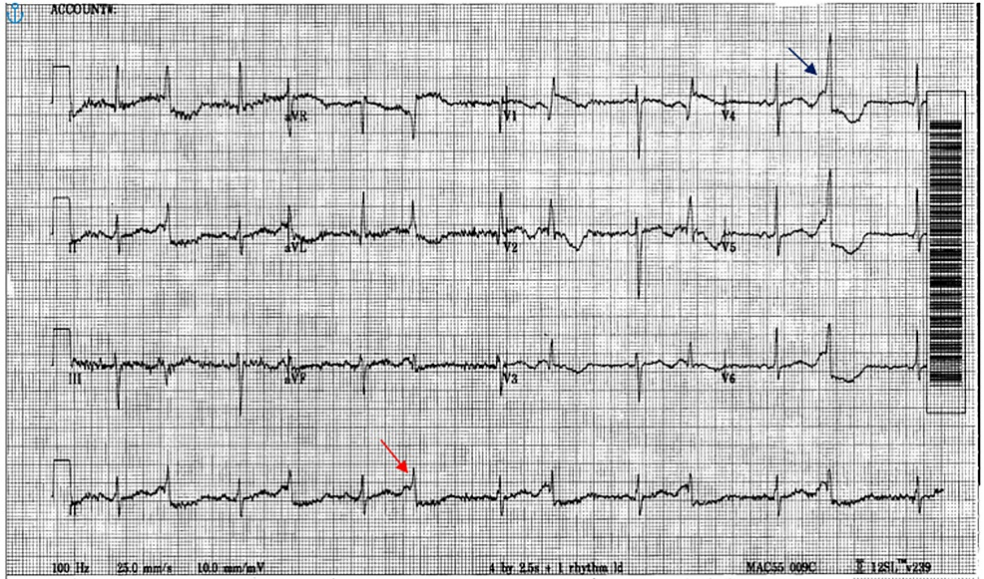
Emergency department EKG showed sinus rhythm with PVC (red and blue arrows), bigeminy, prolonged QT, and non-specific ST/T-wave changes. PVC: premature ventricular contractions

Upon arrival at the emergency department, she was ill-appearing and was in mild to moderate distress. Her vital signs showed a heart rate of 72 beats/min, blood pressure of 176/98 mmHg, respiratory rate of 18 breaths/minute, and temperature of 97.6°F. Her initial laboratory investigations revealed serum sodium of 116 mmol/L, serum potassium of 3.1 mmol/L, magnesium of 1.3 mg/dL, serum osmolality was 251 mOsm/kg, serum high sensitive cardiac troponin (hs-cTn) T 48 and repeat hs-cTn 51, serum creatinine of 0.8 mg/dL, aspartate aminotransaminase of 47 U/mL, and alanine aminotransferase of 55 U/mL; extended urine drug screen was negative, urine osmolality 200 mOsm/kg, and urine sodium 39 mEq/L. On admission, potassium and magnesium were corrected with repeat labs showing potassium of 4.6 mmol/L and magnesium of 2.1 mg/dL. Because she has a low oral intake, the first thought was that her hyponatremia was caused by tea and toast syndrome. EKG showed sinus rhythm with R on T phenomenon, bigeminy, prolonged QT of 551 ms, and non-specific ST/T-wave changes (Figure 1). CT scan of the head without contrast showed no acute intracranial pathology. Carotid duplex ultrasonography showed no hemodynamically significant stenosis.

**Figure 2:**
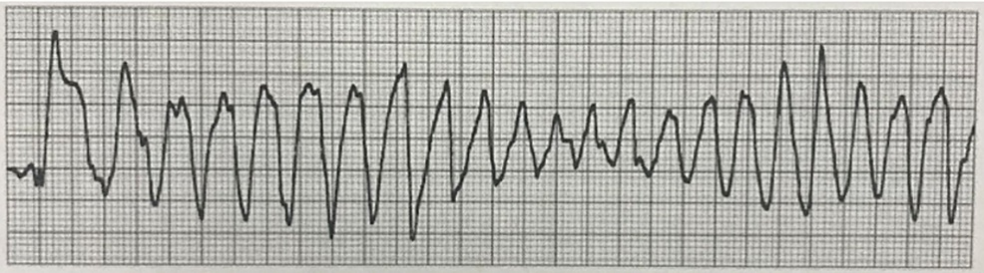
Telemetry rhythm strip showed nonsustained ventricular tachycardia.

The patient was transferred to the intensive care unit, vandetanib was stopped, and her electrolyte was corrected. She received hypertonic saline followed by sodium chloride 3 gm tablets three times a day. Hyponatremia was corrected to 147 mmol/L by the third day. On admission, potassium and magnesium were corrected with repeat tests showing potassium of 4.6 mmol/L and magnesium of 2.1 mg/dL. However, despite the correction of electrolytes, the patient developed TdP, which made the team think of vandetanib as the common source as it can lead to electrolyte abnormalities, QT prolongation, and TdP. Two days after presentation, she developed palpitations, shortness of breath, and nonsustained ventricular tachycardia with a ventricular rate of 210 beats/minute. Telemetry showed runs of nonsustained ventricular tachycardia (Figure 2) and TdP.

**Figure 3:**
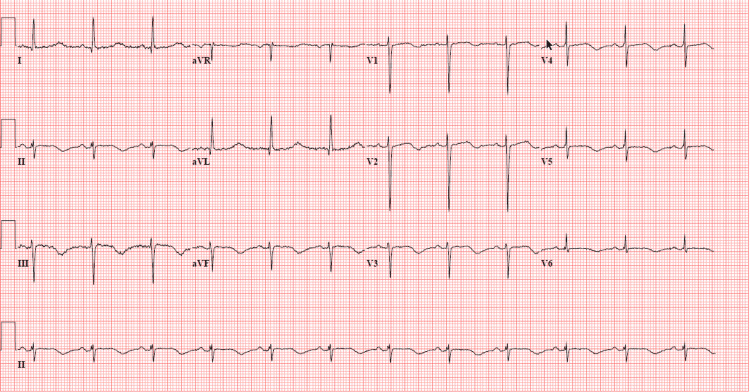
Repeat EKG showed normal sinus rhythm, left axis deviation, minimal voltage criteria for LVH, T wave abnormality, and prolonged QTc interval of 617 ms. LVH: left ventricular hypertrophy

Over five hours, she had two episodes of self-limiting TdP, each lasting less than 20 seconds. She was given 2 gm of magnesium sulfate IV over 15 minutes twice during this period. Repeat EKG showed normal sinus rhythm, left axis deviation, minimal voltage criteria for LVH, T wave abnormality, and prolonged QTc interval of 617 ms in the absence of electrolyte abnormalities, i.e., potassium of 4.3 mmol/L and magnesium of 2.0 mg/dL (Figure 3). Defibrillator pads were placed for the possible need for direct current cardioversion (DCCV). No further ventricular arrhythmias were noted.

Transthoracic echocardiogram showed a borderline left ventricular ejection fraction of 51.4%, diastolic dysfunction, mildly dilated left atrium, trace mitral regurgitation, and mild to moderate aortic regurgitation. Stress echocardiography and echocardiography before the start of vandetanib were unremarkable with normal exercise stress EKG and left ventricular ejection fraction of 67% without significant valvular pathology. Coronary angiography was not considered because she had indeterminate hs-cTn, no chest pain or shortness of breath, and recent normal exercise stress EKG and stress echocardiography. The patient remained hemodynamically stable and was discharged home with a follow-up appointment.

## Discussion

Vandetanib is a receptor TKI that inhibits many targets, including VEGFR2, EGFR, and RET. It has been used to treat various cancers. It is the first drug approved for the treatment of adult patients with unresectable, locally advanced, or metastatic disease in the United States and Europe [6]. However, it has been reported to induce several adverse cardiac effects by unknown underlying mechanisms. [7] Vandetanib is also known to have other side effects, just like other TKIs, such as rash, nausea, diarrhea/colitis, and hypertension in a significant number of patients. In some cases, vandetanib-induced prolonged QTc interval and TdP have been reported; however, very little is known about its electrophysiological effects [8,9]. Zang et al. conducted a systematic review and meta-analysis to understand the risk of QTc interval prolongation among cancer patients taking vandetanib. The review showed that the treatment with vandetanib is associated with a significant increase in the overall incidence and risk of QTc interval prolongation, which can eventually be leading to TdP [9].

TdP is a form of polymorphic ventricular tachycardia that occurs in patients with congenital or acquired QTc interval prolongation, and, usually, the rate varies between 160-250 beats per minute [10]. TdP is usually short-lasting and mostly terminates spontaneously. However, it is not unusual to have multiple episodes of TdP, which can recur in rapid succession and potentially can lead to ventricular fibrillation and sudden cardiac death (SCD) [10,11]. Prolonged QTc increases the risk of TdP, mainly when QTc is more than 500 ms. The risk of TdP also increases when the QTc interval becomes prolonged greater than 60 ms compared with the pretreatment value [12]. Common risk factors for TdP is mentioned in Table 1 [13].

**Table 1:** Risk factors for TdP TdP: torsades de pointes

QTc interval >500 ms
Increase in QTc interval >60 ms compared with pretreatment value
Female sex
Advanced age
Heart failure with reduced ejection fraction
Acute myocardial infarction
Bradycardia
Possible genetic predisposition
Electrolyte abnormalities, such as hypokalemia, hypomagnesemia, and hypocalcemia
Concurrent administration of more than one QTc interval prolonging drugs
Elevated plasma concentrations of QTc interval–prolonging drugs (In acute kidney injury or chronic kidney disease patients, drug interactions or intravenous infusion of QTc prolonging drugs)

**Figure 4:**
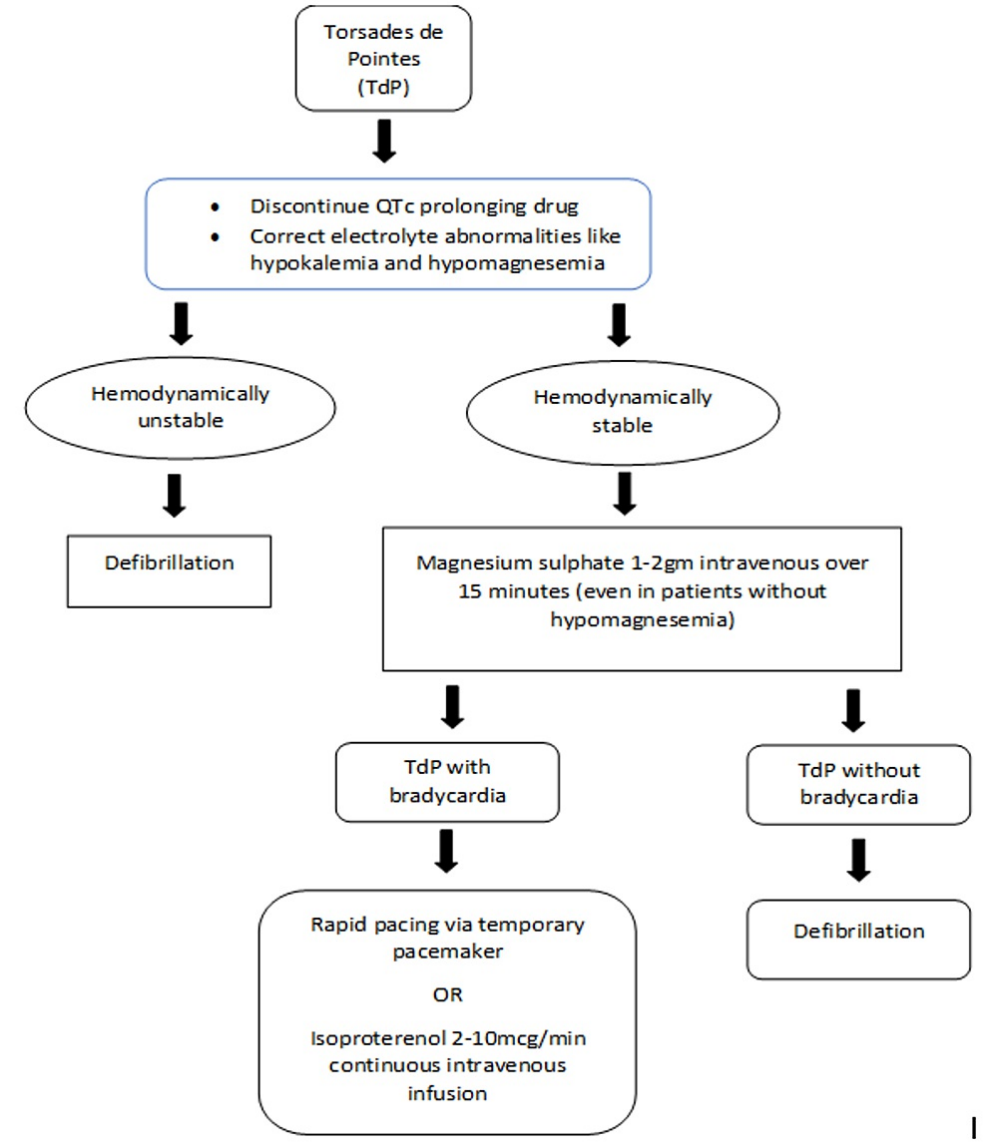
Management algorithm for TdP TdP: torsades de pointes

The risk factors in our patient besides were advanced age, female sex, hypomagnesemia and hypokalemia, and vandetanib induced QTc prolongation. Our patient was having multiple episodes of TdP, which was a potential risk factor for SCD. The management strategies for TdP are described as an algorithm in Figure 4 [13]. As our patient was hemodynamically stable, we considered a trial of intravenous magnesium sulfate, which resolved the episodes of TdP. Later vandetanib was discontinued, and electrolytes were corrected, i.e., potassium at 4.3 mmol/L, sodium at 147 mmol/L, and magnesium at 2.0 mg/dL. The patient was monitored by telemetry, which showed no further episodes of TdP. The patient was referred to an oncologist as an outpatient for the alternative regimen upon discharge.

Vandetanib is associated with several electrolyte disturbances, such as hypocalcemia, hypokalemia, hyponatremia, and hypercalcemia, according to initial studies [14-16]. Patients with hyponatremia have a higher risk of mortality and prolonged hospitalization. Early detection and prompt treatment of this disorder help in preventing serious neurologic complications and thus improve overall survival [17-19]. The mechanism behind vandetanib-induced hyponatremia is not clear. The studies have demonstrated the activity of VEGF in renal sodium metabolism and, thus, the role of anti-VEGF/VEGF receptor antagonists in sodium homeostasis, which may explain the mechanism of hyponatremia in patients using vandetanib [20]. In our patient, decreased appetite, poor oral intake, and excessive water intake due to dry mouth (a side effect of vandetanib) were also contributory factors for hyponatremia besides the use of vandetanib. Hyponatremia improved after discontinuing vandetanib in our case.

## Conclusions

MTC is a neuroendocrine tumor of the thyroid gland with a poor prognosis in advanced stages. Surgery and medical therapy with therapeutic efficacy are promising with significantly prolonged progression-free survival in the patient who is on vandetanib. Careful monitoring for the side effects is equally important as vandetanib therapy is associated with serious cardiovascular side effects, including hypertensive crisis and arrhythmias due to prolonged QTc. Vandetanib should be withheld until QTc is <450 msec, then can resume at a reduced dose.
